# The single-cell opioid responses in the context of HIV (SCORCH) consortium

**DOI:** 10.1038/s41380-024-02620-7

**Published:** 2024-06-15

**Authors:** Seth A. Ament, Rianne R. Campbell, Mary Kay Lobo, Joseph P. Receveur, Kriti Agrawal, Alejandra Borjabad, Siddappa N. Byrareddy, Linda Chang, Declan Clarke, Prashant Emani, Dana Gabuzda, Kyle J. Gaulton, Michelle Giglio, Federico M. Giorgi, Busra Gok, Chittibabu Guda, Eran Hadas, Brian R. Herb, Wen Hu, Anita Huttner, Mohammad R. Ishmam, Michelle M. Jacobs, Jennifer Kelschenbach, Dong-Wook Kim, Cheyu Lee, Shuhui Liu, Xiaokun Liu, Bertha K. Madras, Anup A. Mahurkar, Deborah C. Mash, Eran A. Mukamel, Meng Niu, Richard M. O’Connor, Chelsea M. Pagan, Alina P. S. Pang, Piya Pillai, Vez Repunte-Canonigo, W. Brad Ruzicka, Jay Stanley, Timothy Tickle, Shang-Yi A. Tsai, Allen Wang, Lauren Wills, Alyssa M. Wilson, Susan N. Wright, Siwei Xu, Junchen Yang, Maryam Zand, Le Zhang, Jing Zhang, Schahram Akbarian, Shilpa Buch, Christine S. Cheng, Michael J. Corley, Howard S. Fox, Mark Gerstein, Suryaram Gummuluru, Myriam Heiman, Ya-Chi Ho, Manolis Kellis, Paul J. Kenny, Yuval Kluger, Teresa A. Milner, David J. Moore, Susan Morgello, Lishomwa C. Ndhlovu, Tariq M. Rana, Pietro Paolo Sanna, John S. Satterlee, Nenad Sestan, Stephen A. Spector, Serena Spudich, Hagen U. Tilgner, David J. Volsky, Owen R. White, Dionne W. Williams, Hongkui Zeng

**Affiliations:** 1 University of Maryland School of Medicine, Baltimore, MD USA; 2 Yale School of Medicine, New Haven, CT USA; 3 Icahn School of Medicine at Mount Sinai, New York, NY USA; 4 University of Nebraska Medical Center, Omaha, NE USA; 5 Dana-Farber Cancer Institute, Harvard Medical School, Boston, MA USA; 6 University of California San Diego, La Jolla, CA USA; 7 University of Bologna, Bologna, Italy; 8 Boston University, Boston, MA USA; 9 Weill Cornell Medicine, New York, NY USA; 10 Allen Institute for Brain Science, Seattle, WA USA; 11 University of California Irvine, Irvine, CA USA; 12 McLean Hospital, Harvard Medical School, Belmont, MA USA; 13 University of Miami, Miami, FL USA; 14 The Scripps Research Institute, La Jolla, CA USA; 15 Broad Institute of MIT and Harvard, Cambridge, MA USA; 16 National Institute on Drug Abuse, Bethesda, MD USA; 17 Massachusetts Institute of Technology, Cambridge, MA USA; 18 Emory University School of Medicine, Atlanta, GA USA

**Keywords:** Addiction, Genetics

## Abstract

Substance use disorders (SUD) and drug addiction are major threats to public health, impacting not only the millions of individuals struggling with SUD, but also surrounding families and communities. One of the seminal challenges in treating and studying addiction in human populations is the high prevalence of co-morbid conditions, including an increased risk of contracting a human immunodeficiency virus (HIV) infection. Of the ~15 million people who inject drugs globally, 17% are persons with HIV. Conversely, HIV is a risk factor for SUD because chronic pain syndromes, often encountered in persons with HIV, can lead to an increased use of opioid pain medications that in turn can increase the risk for opioid addiction. We hypothesize that SUD and HIV exert shared effects on brain cell types, including adaptations related to neuroplasticity, neurodegeneration, and neuroinflammation. Basic research is needed to refine our understanding of these affected cell types and adaptations. Studying the effects of SUD in the context of HIV at the single-cell level represents a compelling strategy to understand the reciprocal interactions among both conditions, made feasible by the availability of large, extensively-phenotyped human brain tissue collections that have been amassed by the Neuro-HIV research community. In addition, sophisticated animal models that have been developed for both conditions provide a means to precisely evaluate specific exposures and stages of disease. We propose that single-cell genomics is a uniquely powerful technology to characterize the effects of SUD and HIV in the brain, integrating data from human cohorts and animal models. We have formed the Single-Cell Opioid Responses in the Context of HIV (SCORCH) consortium to carry out this strategy.

## Introduction

Substance use disorders (SUD) represent one of the major public health challenges of our era. An estimated 5% of the world’s population uses illicit drugs, which can lead to devastating personal consequences and a tragically high rate of overdose deaths, as well as enormous economic costs to address the negative impacts of SUD [1]. In particular, the non-medical use of opioids and opioid use disorders (OUD) have increased dramatically throughout the world in recent years [2, 3]. Approximately 61 million people globally used opioids in 2020 [4, 5], with >80,000 opioid overdose deaths in the United States alone in 2021 [6].

A profound challenge both in treating and studying SUDs in human populations is the high prevalence of co-morbid conditions, including polysubstance use, comorbid psychiatric and medical disorders, and the pervasive effects of socioeconomic factors [4]. A particularly vexing issue is the comorbidity of SUD with Human Immunodeficiency Virus-1 (HIV) infection [7]. The HIV pandemic has been an enormous public health concern, having resulted in 75 million HIV infections and 32 million deaths worldwide since the emergence of the epidemic 40 years ago, with profound disparities by region, race, and age [8, 9]. Despite successful suppression of viremia with the advent of combination antiretroviral therapies (cART), ~50% of people with HIV (PWH) experience mostly mild neurocognitive impairments and associated forms of central nervous system dysfunction, which may be related to low-level viral replication in the brain as well as the cART therapies themselves [10]. Of the ~15 million people who inject drugs globally, 17% are PWH [11]. Conversely, it is estimated that up to 84% of PWH may have used at least one addictive substance in their lifetime [12]. Unsafe drug use increases risk of HIV infection. In addition, ~50% of PWH experience chronic pain [13–15] and are more likely to be prescribed opioids at higher doses and for longer periods of time than the general population [16–19]. OUD and problem opioid use, including high-dose opioid therapy and prescription drug misuse, are prevalent among PWH [20–25]. Moreover, substance use in PWH is associated with treatment non-adherence, increased rates of viral transmission, clinical progression of HIV disease, and greater mortality [7, 26–28]. Thus, the negative consequences of SUD and chronic HIV infection continue to be intertwined.

Basic research at the cellular level is needed to reveal the biological effects of both SUD and HIV in the brain and interactions between them. Decades of research into the neurobiological effects of addictive substances have identified three distinct behavioral stages of addiction: binge/intoxication, withdrawal/negative affect, and preoccupation/anticipation resulting in relapse to drug use [29]. These behaviors correspond to the dysregulation of neural circuits related to salience/habits, negative emotional states, and executive function, with the involvement of the basal ganglia, extended amygdala, and prefrontal cortex, respectively [29]. But the critical cell types within these brain regions are not fully described, and the best therapeutic targets within them are not known. Likewise, extensive research has probed the neurobiological mechanisms by which HIV infection in the brain results in neuronal injury and neurocognitive symptoms even in the presence of efficacious cART. Frontostriatal circuits are implicated in HIV neuropathogenesis [10, 30, 31], as in SUD, and the neurotoxic effects of HIV are exacerbated by concomitant opioid exposure, suggesting functional overlap between the two morbidities [32–34]. A better understanding of the neural and molecular mechanisms contributing to these brain abnormalities will be essential to the development of new therapeutics to address the underlying symptoms of both disorders.

Here, we propose that to make progress in understanding the potentially intertwining biological mechanisms of SUD and HIV, we should study them in subjects affected by both morbidities. We describe the scientific and practical rationales for this strategy and how single-cell genomic studies of brain regions from individuals with SUD and HIV, as well as animal models with well-controlled induction of substance use and HIV will help address important knowledge gaps. We have formed the Single-Cell Opioid Responses in the Context of HIV (SCORCH) consortium to implement this strategy.

### Molecular, cellular, and neural circuit mechanisms in SUD

**Fig. 1:**
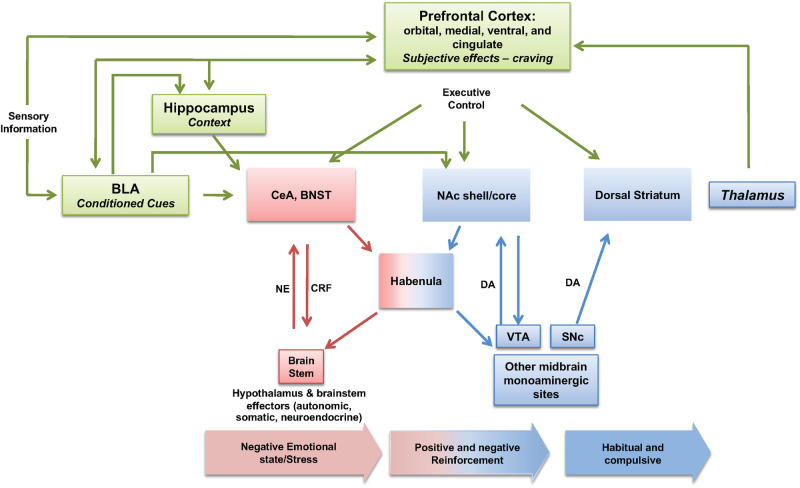
Neural circuitry underlying substance use disorders and addiction. Brain regions: BLA basolateral amygdala, CeA central amygdala, BNST bed nucleus of the stria terminalis, NAc nucleus accumbens, VTA ventral tegmental area, SNc substantia nigra. Neurotransmitters: NE norepinephrine, CRF corticotropin-releasing factor, DA dopamine.

SUDs are associated with changes in behavior that persist long after the cessation of drug use, mediated in part by well-characterized changes in the function of the mesolimbic dopamine (DA) system. Central nodes of this system include the dopaminergic neurons of the ventral tegmental area (VTA) and substantia nigra (SNc), as well as downstream targets such as the nucleus accumbens (NAc), prefrontal cortex (PFC), and extended amygdala circuits (Fig. 1). The activities of many other brain regions are also altered in the context of SUD, including the insular cortex, hippocampus, dorsal striatum, habenula, and thalamic nuclei.

The NAc is a ventral striatal region critical for drug-craving and drug-taking behaviors, as it is considered a gateway for limbic information to engage motor-related circuitry. Dopaminergic neurons from the VTA synapse on two NAc neuronal subtypes: dopamine D_1_ receptor (*DRD1*)-expressing spiny projection neurons (D1R-SPNs) and D_2_ receptor (*DRD2*)-expressing spiny projection neurons (D2R-SPNs). D1R-SPNs and D2R-SPNs have distinct cellular and molecular adaptations to addictive substances [35–38]. Opioids [36, 39] and psychostimulant drugs [39, 40] modify the intrinsic activity of spiny projection neurons (SPNs) and synaptic inputs to these cells. Transcriptional profiling of these cell types has revealed cell type-specific molecular regulators of drug-induced neuroplasticity and behaviors, including actin-binding proteins, epigenetic enzymes, and transcription factors [37, 41–44]. However, as described below, new subtypes of SPNs are now being discovered through single-cell genomics and related techniques, and their differential contributions to SUD are not yet fully understood.

The PFC controls executive functions that become progressively compromised in the context of addiction, leading to impulsivity, perseveration, affective instability, and drug overvaluation. The PFC’s glutamatergic projections to SPNs in the NAc have a major influence on the reinstatement of drug-seeking behavior in rodents [45]. The imbalance of PFC glutamate homeostasis is thought to contribute to drug relapse, as elevated extracellular glutamate levels are seen at PFC-NAc synapses during the resumption of extinguished drug-seeking behaviors, while the depotentiation of this circuit impairs drug-seeking behaviors [46–48]. Long-term abstinence from addictive substances is associated with changes in synaptic plasticity, such as glutamatergic receptor subunit composition and maturation of silent synapses [49, 50].

Addictive substances, including opioids, produce initial intoxication, followed by tolerance and the escalation of intake with continued use. Withdrawal is characterized by negative emotional states, including anxiety, dysphoria, emotional pain, irritability, and sleep disturbances, thereby contributing to compulsive drug seeking and taking [51]. Extended amygdala circuitry, comprising several basal forebrain structures – e.g., bed nucleus of the stria terminalis, central nucleus of the amygdala, and substantia innominata – is key to these negative emotional states and motivation to continue to engage in and escalate drug intake [51]. The central nucleus of the amygdala is crucially involved in the development of morphine withdrawal-induced conditioned place aversion but has less influence on somatic signs of withdrawal [52]. Reciprocal connections have been found between the basolateral amygdala and the hippocampus, sensory association areas, and cortical regions. These reciprocal circuits participate in the encoding and relay of information associated with emotional valence, including drug-related cues [53–56], playing key roles in relapse in animal models of the reinstatement of drug seeking and taking behaviors [55–57].

While much attention has focused on the effects of SUD within neurons, addictive substances also induce changes in glia that contribute to their effects on neural circuits. For instance, astrocytic morphology and expression of the glial glutamate transporter *SLC1A2* (also known as EAAT2 or GLT-1) are affected by exposure to addictive substances [58]. In addition, psychostimulants such as cocaine activate striatal microglia and release of glial cytokines, such as tumor necrosis factor α, which regulate synaptic and behavioral responses to cocaine [59]. Genes related to activity within oligodendrocytes have similarly been affected by various cocaine exposure paradigms.

Persistent cell type-specific changes in gene regulation underlie the altered states of these mesolimbic circuits in addiction. Transcription factors (TFs), such as the truncated form of *FOSB* (ΔFosB), phosphorylated cyclic adenosine monophosphate response element binding protein (pCREB), early growth response protein 1/2/3 (*EGR1/2/3*), and nuclear factor κB (NFκB), regulate the expression of drug-responsive genes [60–65], and altered activity of these TF networks is thought to promote aberrant plasticity and addiction-like behaviors [37, 60, 63, 65, 66]. MicroRNAs and other non-coding RNAs with regulatory functions are also altered in the brain with exposure to addictive substances and can functionally regulate drug intake in animal models [67, 68]. These gene regulatory changes may occur either in parallel or downstream of disrupted signaling cascades regulated by such molecules as the serum/glucocorticoid-regulated protein kinase 1 (*SGK1*) [69] and brain-derived neurotrophic factor (BDNF)-TrkB (encoded by *NTRK2*) [70, 71].

Chronic exposure to addictive substances also leads to long-lasting changes in chromatin states, with causal roles established for several epigenetic regulatory proteins. Post-translational modifications of histones play critical roles in the activity of promoters, enhancers, and other gene regulatory elements. Histone lysine acetylation, enriched in active regulatory regions, is increased in the NAc after chronic drug use [72–76], and enzymes modifying histone acetylation modulate opioid- [43, 75–79] and cocaine- [73, 74, 80–83] induced reward. In contrast, di-methylation at lysine 9 on the histone 3 tail (H3K9me2), associated with transcriptional repression, is reduced, and its demethylase, *EHMT2* (also known as G9a) modulates behavioral responses to opioids and cocaine [84–86]. DNA methylation at CpG sites generally leads to transcriptional silencing. Long-term withdrawal from cocaine leads to time-dependent increases in DNA methylation [87], and emerging evidence suggests similar effects in opioid withdrawal [88, 89]. Perturbation of the enzymes involved in the deposition and maintenance of DNA methylation modulates cocaine-induced behavioral plasticity [88–90]. However, the directionality of drug-induced changes in DNA methylation and correlations with changes in drug-induced gene expression are not fully understood. Further exploration into how drugs alter transcriptional activity within the brain will be critical in understanding how long-term effects in neuroplasticity and behavior occur.

### Molecular, cellular, and neural circuit mechanisms in HIV, HIV-induced CNS dysfunction, and shared mechanisms with SUD

HIV enters the brain early after infection by transmigration of infected monocytes, macrophages, and T cells through the blood-brain barrier, and it remains in the central nervous system (CNS) for life [91–93]. HIV subsequently spreads throughout the brain, establishing persistent infection primarily in CNS-resident myeloid cells, microglia, and perivascular macrophages. Neurons are not considered to be major targets of infection or viral integration. Prior to the introduction of cART, productively infected brain macrophages and microglia were considered to be the main cellular substrates responsible for pathogenesis of HIV encephalitis and dementia in persons with AIDS. Initiation of cART and life-long treatment adherence reduces HIV burdens to residual levels, prevents (or reverses) immunodeficiency, prevents severe HIV-related diseases, and allows people to live with HIV with near-normal life expectancies. Nevertheless, up to 50% of PWH on stable, HIV suppressive cART develop a range of neurological complications [94]. In the cART era, HIV-associated cognitive diseases are largely a spectrum of minimal to moderate neurocognitive impairments (NCI), which are diagnosed with neuropsychological tests and measures of daily activities [94, 95]. The impairments have been referred to as HIV-Associated Neurocognitive Disorders (HAND), HIV-NCI, or NeuroHIV, and they represent the majority of HIV induced CNS dysfunction currently diagnosed in PWH.

The molecular, cellular, and neural circuit mechanisms of HIV-induced CNS dysfunction are not fully understood. These dysfunctions occur even in the presence of low virus burden in the CNS, estimated in some studies at 4000 viral copies/gram brain tissue [96], and absent the major neuropathologies seen in HIV brain diseases prior to cART introduction [95, 96]. HIV’s persistence in the body in the presence of cART occurs via cells that harbor genome-integrated HIV proviral DNA, which allow the virus to persist and replicate despite cART’s blockade of the transmission of virions to uninfected cells. The brain has unique features to serve as an HIV reservoir. The blood-brain barrier prevents efficient drug penetration of many antiretroviral drugs. Also, brain-resident immune cells have reduced immune surveillance and poor viral genetic information exchange compared to peripheral immune cells. In the brain, HIV DNA and RNA have been detected primarily in brain macrophages/microglia, and to a much lesser extent, astrocytes. However, brain immune cells exist in diverse homeostatic and reactive states [97]. The precise identities, characteristics, and distribution within the brain of these infected cells are uncertain, as are the neural mechanisms by which infected cells mediate HIV-induced CNS dysfunction.

Recent findings indicate that HIV-induced cognitive impairments are associated with persistent low-level neuroimmune activation, suggesting that HIV disrupts CNS immune homeostasis. The induction of blood-brain barrier damage, neuroinflammation, and neuronal injury by brain-resident HIV are proposed to occur through both indirect and direct mechanisms [91, 98]. In the direct mechanism, cellular injury is induced by soluble viral proteins such as Tat, Nef, Vpr, and gp120 acting at a distance from infected cells by interacting with neuronal cell surface proteins, such as *N*-methyl-D-aspartate (NMDA) and chemokine receptors [99]. In the indirect mechanism, infected and/or activated immune cells and astrocytes release neurotoxic/neuroinflammatory factors [100], including reactive oxygen species, cytokines, and glutamate, that induce apoptosis within neighboring neurons [101]. Astrocytes in the brains of PWH may alter glutamatergic-related plasticity, as there are reports of reduced functioning of excitatory amino acid transporters *SLC1A2* and *SLC1A3* (also known as EAAT2 and EAAT1, respectively) following exposure to the HIV Tat protein product [102].

The downstream impacts of HIV-induced neuroinflammation and neuroreactive viral proteins within neural systems remain controversial [91, 96, 99]. Microglial infection results in downregulation of genes related to neuronal support and synaptic regulation, including in PWH without active viral replication in the brain [103]. The dopaminergic system is heavily impacted, with the striatum and substantia nigra exhibiting high amounts of inflammation and pathology [104–106]. Reductions of dopamine in cerebrospinal fluid and of dopaminergic signaling in the brain correlate with worse cognitive symptoms [107, 108]. The serotonergic system is also impacted. Expression of the serotonin transporter (SERT, encoded by *SLC6A4*) is upregulated, likely leading to decreased synaptic levels of serotonin in the brains of simian HIV-infected rhesus macaques, which are infected with a simian-human hybrid immunodeficiency virus, as well as in PWH [109, 110]. PWH are twice as likely to be diagnosed with major depressive disorder and are responsive to serotonin reuptake inhibitors [110, 111]. The underlying mechanisms driving these changes in dopaminergic and serotonergic activity remain unclear.

Although transcriptional regulators of HIV integration are well-characterized in peripheral blood, those responsible for brain integration and potentially CNS symptoms remain poorly characterized. In terms of infection, HIV is an RNA virus that undergoes reverse transcription to convert into a DNA provirus. These proviruses are packaged by nucleosomes and regulated by host and viral transcription factors. Studies performed primarily in peripheral immune cells have shown that the activity of epigenetic enzymes and transcription factors, such as Tat, are crucial for both integration into the host genome and latent stages following cART [112]. This includes histone acetylation, which facilitates HIV integration [113], and histone methyltransferases such as *EHMT1* and *EZH2*, which are implicated in HIV viral latency mechanisms [114, 115]. Under cART, HIV can enter latent stages through transcriptional silencing, which allows the virus to continue to exist, then at later times undergo reactivation via activity of transcription factors such as NFκB [116]. Epigenetic mechanisms are proposed to allow for HIV persistence following cART [117], yet the precise mechanisms at play in HIV persistence and HIV-induced CNS dysfunction following cART are unknown. Of note, the molecular mechanisms of HIV latency in brain immune cells may differ from what has been described in peripheral T cells [118]. New studies are needed to characterize the chromatin landscape within HIV+ tissues, and in particular brain, to reveal the transcriptional regulatory mechanisms underlying HIV infection and persistence.

The interactions between HIV and SUD pathophysiologies are complex, and substance use in PWH is associated with worse clinical outcomes. Efforts to dissect the effects of these conditions in the human CNS have employed neuroimaging, cerebrospinal fluid, and blood biomarkers, and neuropsychiatric assessments. However, there is a paucity of knowledge of how drug use changes the course of HIV pathogenesis and latency, and several mechanisms have been proposed. Some plausible mechanisms involve the shared effects of SUD and HIV and induced CNS dysfunction on neuroinflammation [119–121], which may be exacerbated by interactions between opioids and HIV proteins that affect the functioning of neurons [122–124], astrocytes [121, 123, 125–127], and/or microglia [123, 128–130]. Opioids may increase HIV pathogenesis by increasing the number of infected circulating monocytes [131]. This likely occurs via a dopamine-dependent mechanism, as monocytes express dopamine receptors and are responsive to dopamine receptor agonists [132]. Opioids may also directly impact HIV infection and neuroinflammation through opioid receptors, which are expressed by many cell types. Monocytes, microglia, and astrocytes express all three opioid receptor subtypes: mu, kappa, and delta [133, 134]. Addictive substances may also influence viral replication by altering the activity of transcription factors, such as CREB, that are both affected by addictive substances and part of signaling systems that promote HIV replication [135]. However, it is unknown whether opioid-related mechanisms converge with and promote HIV neuropathology. Studies to identify common molecules within the above-described brain regions and cell types are needed to understand how the interaction of opioids and HIV influences pathogenesis,

### Insights into SUD and HIV from single-cell genomics

Epigenetic regulation plays important roles in both SUD and the persistence of HIV in the brain. However, the cell type-specific transcriptional and epigenomic mechanisms underlying disease-associated neuroinflammation and neuroplasticity are unclear. Single-cell genomics, along with emerging spatial transcriptomics technologies, have given researchers a powerful new approach to profile the inner workings of cellular subtype mechanisms, both in human post-mortem brain tissue and in animal models. These technologies have been used to identify and classify sub-populations of neurons and glia [136–138], and trace cellular dynamics and gene expression across cell states, including disease progression [139–141]. The neuroscience field has had difficulty with isolating intact whole single-cells, especially from frozen brain tissue, as neurons are morphologically complex with long axons and dendritic processes. Fortunately, isolating cellular nuclei from frozen brain tissues for single-nucleus genomic profiling has proven to be a viable alternative and has been swiftly implemented within the field. Importantly, gene expression profiles derived from neuronal nuclei vs. whole cells are highly concordant [142]. Single-nucleus RNA sequencing (snRNA-seq) in frozen tissue has been shown to be successful in profiling synaptic [143–146] and microglial [139, 141, 147] transcripts and decouples tissue dissection from sample processing [148].

Single-cell and spatial genomic studies have already made substantial inroads to elucidate the diversity of cell types in cortical and subcortical brain regions relevant to SUD and HIV [136, 137, 149–152]. snRNA-seq has revealed hundreds of transcriptionally distinct cell populations in these brain regions. Single-cell epigenomic profiling of chromatin accessibility (scATAC-seq) and DNA CpG methylation (scMethyl-seq) have revealed cell type-specific regulatory elements [142, 153, 154]. Spatial transcriptomics and multimodal characterization demonstrate that most of these transcriptomic populations correspond to bona fide cell types with distinct spatial distributions and morphological and physiological characteristics [137].

An illustrative example is the spiny projection neurons of the striatum. SPNs represent >90% of striatal neurons. As noted above, there are two major SPN populations that express dopamine D_1_ and D_2_ receptors, respectively. In addition, SPNs located in different striatal subregions have distinct projection patterns and physiological characteristics. However, the molecular identities of these cells were not well understood. Single-cell and spatial profiling have revealed at least 10-20 transcriptionally distinct SPN subtypes [136, 150, 152, 155–158]. These studies defined molecular markers for discrete subtypes of D_1_ and D_2_ SPNs, including in striosome vs. matrix subcompartments of the dorsal striatum [159], as well as in core vs. shell regions of the nucleus accumbens [152]. Additional markers defined continuous variation in SPNs along the dorsoventral and rostrocaudal axes [150, 152]. Surprisingly, many studies revealed that a substantial proportion of SPNs have atypical characteristics, such as co-expression of D_1_ and D_2_ receptors, along with additional markers that distinguish them from canonical SPN subtypes [136, 150, 152]. Single-cell epigenomic data are beginning to elucidate the regulatory networks contributing to SPN diversity and will likely enable the development of new genetic tools for functional perturbation of specific SPN subtypes [156, 157, 160]. We anticipate that higher resolution molecularly-defined SPN subtypes will have distinct functions in SUD and HIV, extending previous work on subtypes defined by other approaches. Despite this progress, a substantial issue is the lack of a consensus atlas for SPN subtypes, and it is unclear how reproducible these subtypes will be across conditions and species. Arriving at a consensus on these issues will be necessary to enable comparisons across studies [161]. At present, snRNA-seq atlases for the diversity of neurons in some other brain regions relevant to SUD and HIV remain rudimentary.

Single-cell genomics studies have begun to profile the molecular changes in brain cell types in the context of SUD and HIV. Studies related to SUD have used human post-mortem brain tissue [141, 149, 162], animal models [163–167], and human pluripotent stem cells [168–170]. These studies provide several insights. First, gene expression changes associated with drug exposure are detected in all major neuronal and non-neuronal cell types, suggesting widespread effects in the brain. Second, most studies have detected changes in transcriptional signatures related to neuroinflammation. Third, changes in the expression of synaptic genes are detected in certain neuronal subtypes, perhaps describing forms of neuroplasticity. Fourth, transcription factors and chromatin remodeling factors are enriched among cell type-specific differentially expressed genes, providing insights into the regulation of these neuroinflammatory and neuroplasticity gene signatures.

Single-cell genomic studies on the effects of HIV in the brain initially focused on the roles of brain immune cells [103, 165, 171–175]. These studies provided insight into the brain cell types that serve as persistent reservoirs for HIV in the presence of cART, as well as characterizing the specific reactive and inflammatory states associated with infection. Broader profiling of the CNS is beginning to emerge, including interactions between infection and addictive substances [103, 165]. Unbiased profiling of transcriptional changes in brain cell types will provide insight into the mechanisms of neurodegeneration, as well as transcriptional correlates of neurocognitive and neuropsychiatric phenotypes.

These initial single-cell genomic studies of SUD and HIV demonstrate promise. However, most studies have been done in animal and cell models, as well as in the cerebrospinal fluid and other accessible tissues of PWH. The specific neuroinflammatory, synaptic, and gene regulatory changes appear to be context-specific, varying across brain regions, cell types, drugs, and stages of addiction, and the diversity of these conditions has not been adequately sampled. Large coordinated studies in both humans and model systems are needed to overcome these limitations and arrive at rigorous conclusions.

### The SCORCH Consortium: single-cell genomic studies of SUD and HIV in humans and model systems

There remain substantial gaps in our understanding of how SUD and HIV alter biological processes in the brain. Much of what we know about these effects has been learned through experimental studies in animal and cellular models. It is essential to study the human condition, as models do not perfectly recapitulate the human brain nor the real-world conditions of SUD and HIV. Studying the human condition, however, has inherent challenges. This is, in part, due to the wide-ranging symptoms from HIV and SUD, which include variable patterns of behavioral and neurocognitive impairments, peripheral and neural immune suppression, and altered pain responses. A variety of factors exacerbate these symptoms, related to the onset of drug use/escalation throughout HIV disease progression and type of drug use (i.e., drug class and polydrug use) [51]. Epidemiological studies have identified genetic and environmental risk factors [176–178]. The most severe effects of SUD are observed in only ~10% of PWH who use addictive substances. Similarly, the more severe manifestations of HIV-induced neurocognitive dysfunction are currently observed in <5% of PWH, suggesting substantial variation and resilience in many individuals. In both SUD and HIV, it remains largely unknown how brain circuits mediate the effects of these exposures and risk factors. It is difficult for researchers to properly control for these factors across cohorts in human studies, so parallel studies are needed to comprehensively model these variables in tractable animal models of SUD and HIV-induced CNS dysfunction [179, 180] and to correlate findings from these models with the patterns detected in humans. Overall, given the rampant escalation of drug use in the United States and high prevalence of HIV in intravenous drug users, further investigation of the synergistic and long-term effects of HIV and SUD in humans remain an urgent public health priority.

Here, we propose that studying SUD and HIV together represents a promising strategy to understand both disorders. SUD and HIV influence many of the same biological systems, including shared effects on neural circuits related to reward and cognition, persistent neuroinflammation, shared effects on neurotransmitter systems (e.g., dopaminergic signaling), and preliminary evidence of shared effects on transcriptional regulatory programs. Since these mechanisms at least partially overlap between the two disorders, studies designed to investigate these conditions together can provide key insights into both disorders. In addition, we believe that understanding the interactions between these two conditions will provide generalizable insights into the mechanisms underlying clinical variation more broadly. Thus, this strategy will provide benefits not only for understanding the interactions between SUD and HIV, but also for understanding their effects more broadly.

Studies of SUD in the context of HIV also benefit from unique brain tissue resources developed by the Neuro-HIV research community. The National NeuroAIDS Tissue Consortium (NNTC) was established in 1998 to collect, store, and distribute biosamples collected from PWH, as well as unaffected individuals, to support researchers around the world and further our knowledge of nervous system disorders resulting from HIV infection [181, 182]. The NNTC’s unique contribution is its well-characterized and high-quality specimens. Extensive efforts have been made to collect comprehensive neuromedical, neuropsychological, and psychiatric data prior to death. Detailed pathological evaluations of brain, spinal cord, and peripheral organs are conducted post-mortem, and samples are then banked according to strict established protocols to ensure uniformity across the four clinical sites. As of November 2, 2022, 3322 participants had enrolled in the clinical evaluation/tissue donation program, and 2,303 individuals have donated CNS material to the bank. Psychiatric evaluations indicate 381 participants with a lifetime history of opioid use/dependence, as well as 892 with cocaine dependence, 193 for hallucinogens, 237 for sedatives, 468 for stimulants, and 1112 for alcohol. These numbers are nonexclusive, as polysubstance use is not uncommon. Additional criteria are available to determine which of these participants may be most suitable to build cohorts for studies of SUD in the context of HIV. Resources from the NNTC complement other brain banks focused specifically on SUD, which have smaller numbers of samples but are more strongly enriched for severe cases and for donors who died from drug overdose. Additional brain banks have donor tissue without HIV or SUD, as well as with or without other conditions.

**Fig. 2:**
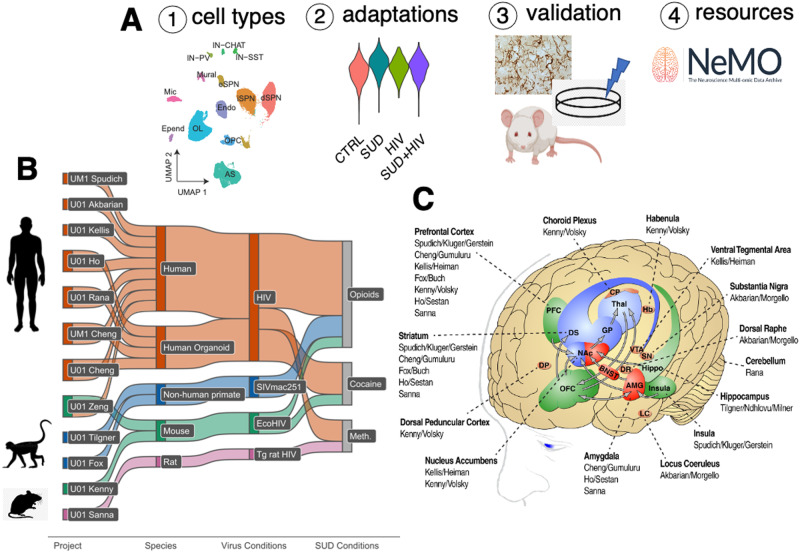
The Single-cell opioid responses in the context of HIV (SCORCH) consortium. **A** The SCORCH consortium will characterize the effects of SUD and HIV in the brain by elucidating the diversity of cell types (1) and cell type-specific molecular adaptations (2) via single-cell genomics in multiple brain regions of humans, non-human primates, and rodents. Key results will be validated through orthogonal approaches, including spatial transcriptomic profiling of brain tissues and genetic perturbation studies in human organoids and rodent models. (3). Data resources from these experiments will be publicly available through the SCORCH Data Center (4). **B** Data will be collected across 12 projects using brain tissue from humans, non-human primates, and rodent models. **C** Collectively, SCORCH researchers will investigate >15 brain regions relevant to SUD and HIV.

The SCORCH consortium was formed by the National Institute on Drug Abuse in 2020 to support collaborative genomic studies of SUD and HIV (Fig. 2A). The primary strategy of the SCORCH consortium is to gain insights into cellular and molecular mechanisms of SUD, HIV, and SUD + HIV by generating single-cell transcriptomic and epigenomic data in affected brain regions from hundreds of human donors, as well as from specialized animal models. These data will be analyzed to characterize the diversity of cell types in each of the affected brain regions, catalog the cell type-specific changes in gene expression and chromatin accessibility, and compare these effects to related neurodegenerative and neuropsychiatric conditions. Cellular and molecular targets will then be validated through various methods, including CRISPR technology, organoids, and in situ hybridization.

The first goal of SCORCH analyses will be to classify the diversity of cell types in each brain region relevant to SUD and HIV. As the literature suggests changes in numerous brain regions, our studies collectively will characterize more than 15 brain regions (Fig. 2B). Of these, we will focus most intensively on several nodes in the reward system that are strongly implicated in SUD, including sub-regions of PFC (specifically, Brodmann Area 9), dorsal and ventral striatum (including NAc), and amygdala, all of which will be characterized by multiple groups utilizing human brain tissue, as well as both non-human primate and rodent models. Data from large cohorts will be required — especially from human samples — to overcome the inherent heterogeneity of post-mortem brain tissue samples with respect to tissue quality, as well as biological variation due to disease states, covariates such as age and sex, and other factors. SCORCH intends to generate datasets on the scale of a million cells or more from each of the targeted brain regions. Existing atlases of cell types in these regions range widely in their maturity, both as reconstructed from single-cell genomics, as well as more traditional anatomical and functional approaches. Robust, large-scale analyses will require consistent naming conventions with corresponding consistency in the use of diagnostic marker genes. Therefore, we will partner with other researchers, including the BRAIN Initiative Cell Atlas Network, to derive consensus models for the cell types in each of these brain regions.

These comparative cell type atlases will be facilitated by state-of-the-art approaches for single-cell genomic data integration. Recent analyses by BRAIN Initiative researchers have demonstrated the feasibility of combining single-cell genomic data across species to compare brain cell types at fine resolution [183]. Alternative approaches compare cell types based on shared marker genes without requiring direct integration [184]. Leaning on reference atlases from non-diseased individuals will help ensure the robustness of cell type annotations. Both SCORCH-produced and external reference atlases will also provide complementary information about the spatial positioning of cell types within brain regions, as well as neuronal morphology, projection patterns, and physiological properties [137, 185–187].

The second goal of SCORCH analyses will be to characterize the gene expression and chromatin accessibility changes in each cell type within the affected brain regions. We hypothesize that many of these changes will relate to neuroinflammation, neuroplasticity, and gene regulatory processes, building on previous studies. To elucidate these disease processes, we will interpret our data in the context of gene networks reconstructed from our data and prior knowledge about the functional interactions among genes. An important component of our studies will be to compare data from human cohorts with SUD and HIV to data from animal models generated both within and outside the consortium. Animal models allow experimental evaluation of variables such as the effects of single versus multidrug use, drug doses, age, sex, and time of drug administration versus time of HIV infection. For instance, we may find distinct signatures of injected vs. prescription opioids, which have different underlying psychopathologies. SCORCH researchers will investigate both opioid drugs and stimulants such as methamphetamines and cocaine. As noted above, all of these drugs have long-lasting effects on neural circuits related to addiction, likely involving both shared and distinct molecular signatures. The most robust gene associations across human and animal model datasets will be revealed via meta-analyses. We will also perform comparative analyses to assess similarities and differences of the cell type-specific gene expression and chromatin accessibility changes in SUD and HIV vs. other neurodegenerative and neuropsychiatric conditions. In particular, snRNA-seq of neocortical regions is available from large cohorts with Alzheimer’s disease [188, 189], schizophrenia [190], autism spectrum disorders [140, 191], and mood disorders [192], all of which may involve shared molecular signatures with SUD and HIV [193]. Cell type-specific expression quantitative trait loci derived from our data will be used to fine-map risk loci from genome-wide association studies of HIV-induced CNS dysfunction and SUD.

We will consider diverse approaches to elucidate biological signatures of exposure to addictive substances and HIV infection and to compare them across conditions and species. Similar analyses have recently been undertaken to compare gene expression dynamics during brain development across species and brain regions. Successful strategies for this included meta-analyses of gene co-expression modules [194], as well as structured joint decomposition and transfer learning techniques [195]. Similarly, we can identify a gene signature of substance use in one condition (say, a well-controlled animal model) then project that signature into datasets from humans with SUD. Gene signatures can also be interpreted with respect to biological processes such as neuroinflammation or activity-dependent gene expression.

Validation experiments will complement these sequencing data, including examining brain tissue from these and other cases for cell- and site-specific expression patterns of differentially expressed genes by in situ hybridization, immunohistochemistry, or spatial transcriptomics. Our investigators will also study the top differentially expressed genes using human pluripotent-stem cell-derived neuronal culture systems, in combination with in vitro HIV infection and/or stimulation with addictive substances. At a more physiological level, validation experiments will be conducted in suitable animal models of HIV-induced CNS dysfunction and drug administration. In rodent, selected genes identified in our studies can be manipulated using cell subtype-specific genetic tools, including viral and transgenic vectors. Promising molecules could be targeted with pharmacological approaches for translation back to clinical populations. We expect these results will facilitate the identification and functional characterization of cellular circuits and cell type-specific transcripts related to SUD in the context of HIV infection, CNS dysfunction, and its subclassifications.

Finally, a fundamental goal of the SCORCH consortium is to make data resources available to the broader research community, consistent with data FAIRness (Findable, Accessible, Interoperable, Reusable) [196]. We intend to make both raw and processed data available in a timely fashion through the SCORCH Data Center, including metadata required for secondary analyses. In addition, we intend to develop SCORCH web resources (scorch.igs.umaryland.edu), building on an existing web portal to search and access single-cell genomic datasets (nemoarchive.org), cloud-computing environment for large-scale data processing using consensus pipelines (terra.bio), and tools for web-based data visualization and analysis (nemoanalytics.org) [197, 198]. Harmonized molecular and single cell HIV/SUD data sets will enable data mining by the scientific community to identify HIV and/or SUD biomarkers and identify candidate pathways for therapeutic intervention.

Advancing our knowledge of the interactions between SUD, HIV infection and HIV induced CNS dysfunction, and the underlying mechanisms requires this precise transcriptome and chromatin cellular resolution that can be integrated with available SUD and HIV and single-cell datasets. The SCORCH collaborative research teams are uniquely poised to achieve these goals and advance the field toward enhanced mechanistic knowledge that has translational impact on SUD and HIV clinical populations. Overall, the SCORCH consortium seeks to address, at the single-cell level, critical gaps in our understanding of the molecular and cellular perturbations in the brain in individuals with SUDs, HIV infection, and a confluence of these conditions.
